# Supplementary material for: Patterns of Intron Gain and Loss in Fungi
Source: PLoS Biol. 2004 Nov 30;2(12):e422. doi: 10.1371/journal.pbio.0020422 (PMC532390; doi:10.1371/journal.pbio.0020422)
Supplement: Table S1 — Also available at http://genes.mit.edu/NielsenEtAl/. (4.3 MB ZIP). [file pbio.0020422.st001.zip › NielsenEtAl/html/866.html]

AN1222.1.NCU02657.1.MG00383.1.FG00421.1


```
 CLUSTAL W (1.82) Multiple Sequence Alignments - Introns Inserted


Sequence 1: NCU02657.1	395 aa
Sequence 2: MG00383.1	400 aa
Sequence 3: FG00421.1	402 aa
Sequence 4: AN1222.1	388 aa
Alignment Length: 408 aa
Number Identitical Residues: 302 aa
Alignment Score (without introns) 12704


MG00383.1 	MAPNGVNG-------ARH-NEGTFLFT~DD2GSKQSESVGEGHPDKIADQVSDAVLDA~C
NCU02657.1	MATNGVNG-------AKHYNEGTFLFT0--~----SESVGEGHPDKIADQVSDAILDA~C
FG00421.1 	MASNGTNGVHADPASWKHYNEGSFLFT0--~----SESVGEGHPDKIADQVSDAILDA~C
AN1222.1  	MGSVATPN-------------GTFLFT~--~----SESVGRGHPDKIADQISDAILDA1C
          	*.. .. .             *:****        *****.*********:***:*** *

MG00383.1 	LAQDPLSKVACETATKTG~MIMIFGEITTKAKVDFQKAVREAIKDIGYDDSSKGFDYKTC
NCU02657.1	LAEDPLSKVACETATKTG~MIMVFGEITTKAKLDYQKVVRNAIKDIGYDDSSKGFDYKTC
FG00421.1 	LREDPLSKVACETATKTG~MIMVFGEITTQAKLDYQKVVRDAIKDIGYDDSAKGFDYKTC
AN1222.1  	LAEDPLSKVACETATKTG1MIMVFGEITTQARLDYQAIIRGAIKDIGYDDSEKGFDYKTC
          	* :*************** ***:******:*::*:*  :* ********** ********

MG00383.1 	NLLIAIEEQSPDIAQGLHYEKALEELGAGDQGIMFGYATDETPELFPMTLLLAHQLNAAM
NCU02657.1	NLLVAIEEQSPDIAQGLHLDDRLENLGAGDQGIMFGYATDETPELFPLTLLFAHKLNAAM
FG00421.1 	NLLVAIEQQSPDIAQGLHYEKALEQLGAGDQGIMFGYATDETPELFPLTLLFAHKLNAAM
AN1222.1  	NVLVAIEQQSPDIAQGLHYEEALEKLGAGDQGIMFGYATDETPELLPLTLVLSHKLNAAM
          	*:*:***:********** :. **:********************:*:**:::*:*****

MG00383.1 	SA~ARRDGSLPWLRPDTKTQVTIEYKHDNGAVVPLRVDTVVVSAQHSEAITTEQLRKEIL
NCU02657.1	SA~ARRDGSLPWLRPDTKTQVTIEYKHDNGAVVPLRVDTVVVSAQHAPEITTEELRKEIL
FG00421.1 	SA1ARRDGSLPWLRPDTKTQVTIEYKHDNGAVVPQRVHTVVVSAQHSPDITTEELRKEIK
AN1222.1  	TT~ARNDGSIPWLRPDTKTQVTVEYAHDNGAVKPLRVDTVVVSAQHSDDVTTEELRAVIK
          	:: **.***:************:** ****** * **.********:  :***:**  * 

MG00383.1 	EKIIKKVIPSNMLDDKTVYH0IQPSGLFIIGGPQGDAGLTGRKIIVDTYGGWGAHGGGAF
NCU02657.1	EKIIKTTIPAKYLDEKTVYH0IQPSGLFVIGGPQGDAGLTGRKIIVDTYGGWGAHGGGAF
FG00421.1 	EKIIQKVIPAKYLSDETIYH~IQPSGLFIIGGPQGDAGLTGRKIIVDTYGGWGAHGGGAF
AN1222.1  	EKIIKKVIPAELLDDRTVYH~IQPSGRFVIGGPQGDAGLTGRKIIVDTYGGWGAHGGGAF
          	****:..**:: *.:.*:** ***** *:*******************************

MG00383.1 	SGKDFSKVDRSAAYLGRWIAKSLVAAGLARRALVQLSYAIGVAEPLSIYVDTYGTSEKTS
NCU02657.1	SGKDFSKVDRSAAYVGRWIAKSLVAAGLARRALVQLSYAIGVAEPLSIYVDTYGTSDKTS
FG00421.1 	SGKDFSKVDRSAAYVGRWIAKSLVAAGLCRRALVQLSYAIGVAEPLSIYVDSYGTSEKTS
AN1222.1  	SGKDYSKVDRSAAYVGRWIAKSLVAAGLARRALVQLSYAIGVAEPLSLYVDTYGTSEKTS
          	****:*********:*************.******************:***:****:***

MG00383.1 	EELVQIIRDNFDMKPGVIVKELDLAKPIYRQTAKNGHFGTNQSFSWEKPKALKF
NCU02657.1	EELVQIIRNNFDLRPGVIVKELDLAKPIYLQTAKNGHFGTNQSFSWEKPKALKF
FG00421.1 	EELVEIIRKNFDLRPGVIVRELNLDQPIYLQTAKNGHFGTNQSFSWEKPKELKF
AN1222.1  	EELVQIIRNNFDLRPGVIVRELDLAKPIYFETAQNGHF-TNQKFSWEQPKALKF
          	****:***.***::*****:**:* :*** :**:**** ***.****:** ***
```
